# The learning curve for retrograde intrarenal surgery: A prospective analysis

**DOI:** 10.1590/0100-6991e-20223264-en

**Published:** 2022-08-01

**Authors:** THIAGO HENRIQUE CAETANO DA SILVA, CARLO CAMARGO PASSEROTTI, JOSÉ PONTES, LINDA FERREIRA MAXIMIANO, JOSÉ PINHATA OTOCH, JOSE ARNALDO SHIOMI DA CRUZ

**Affiliations:** 1 - Hospital Alemão Oswaldo Cruz, Departamento de Urologia - São Paulo - SP - Brasil; 2 - Faculdade de Medicina da Universidade de São Paulo, Disciplina de Técnica Cirúrgica e Cirurgia Experimental - São Paulo - SP - Brasil; 3 - Hospital Universitário da Universidade de São Paulo - São Paulo - SP - Brasil

**Keywords:** Ureteroscopy, Learning Curve, Nephrolithiasis, Ureteroscopia, Curva de Aprendizado, Nefrolitíase

## Abstract

**Introduction:**

retrograde intrarenal surgery (CRIR) is an evolving tool. Its learning curve is not well established, despite the common use of flexible ureteroscopes today. Our aim is to estimate the number of procedures needed for one to perform RIRS consistently.

**Material and Methods:**

a urology resident had his first 80 RIRS for nephrolithiasis analyzed quantitatively and qualitatively. The procedures were divided into 4 groups containing 20 surgeries each (I to IV), according to their order, for comparison.

**Results:**

there was no difference in stone sizes between groups. All qualitative variables varied significantly between groups (p<0.001), except between III and IV. In the quantitative analysis, there was a difference between groups I and IV in time for double-J catheter placement (p=0.012). There was an increasing difference in sheath placement time (p<0.001) and in total operative time (p=0.004). The time fot stone treatment (p=0.011) was significant only between groups I, II and III. There was difference in total sheath time only between groups I and III (p=0.023). Stone free status did not change between groups.

**Discussion:**

the differences between the qualitative and quantitative variables show the relation between number of surgeries performed and proficiency in the procedure. Intergroup comparisons show sequential optimization of parameters.

**Conclusions:**

we found that 60 is a reasonable number of surgeries to be performed in order to reach the plateau of RIRSs learning curve.

## RESUMO

**Introdução:**

a cirurgia retrógrada intrarrenal (CRIR) é ferramenta em evolução. Sua curva de aprendizado não está bem estabelecida, apesar do uso comum dos ureteroscópios flexíveis atualmente. O objetivo é estimar o número de procedimentos necessários para se realizar CRIR consistentemente.

**Material e Métodos:**

Um residente de urologia teve suas primeiras 80 CRIR para tratamento de nefrolitíase analisadas quantitativa e qualitativamente. Os procedimentos foram divididos em 4 grupos contendo 20 cirurgias cada (I a IV), de acordo com sua ordem, para comparação.

**Resultados:**

Não houve diferença nos tamanhos dos cálculos entre grupos. Todas as variáveis qualitativas apresentaram variação significativa entre os grupos (p<0,001), exceto entre III e IV. Na análise quantitativa houve diferença entre os grupos I e IV no tempo de colocação do cateter duplo J (p=0,012). Houve uma diferença crescente no tempo de colocação da bainha (p<0,001) e no tempo operatório total (p=0,004). O tempo para o tratamento do cálculo (p=0,011) foi significativo apenas entre os grupos I, II e III. Houve diferença no tempo total de bainha apenas entre os grupos I e III (p=0,023). Taxa livre de cálculos não se alterou entre os grupos.

**Discussão:**

as diferenças observadas entres as variáveis qualitativas e quantitativas evidenciam a relação entre o número de cirurgias realizadas e a proficiência no procedimento. As comparações intergrupo mostram otimização sequencial dos parâmetros.

**Conclusões:**

estima-se que 60 é um número razoável de cirurgias para que se atinja o platô da curva de aprendizado.

## INTRODUCTION

In its early years, the use of ureteroscopy was initially limited to diagnostic evaluation of the distal ureter. However, the development and refinement of flexible ureteroscopes made virtually all areas of the urinary tract accessible^1^. Despite the versatility of modern ureteroscopes, the treatment of kidney stones remains the most common indication for the use of ureteroscopic techniques.

Currently, the targets of retrograde intrarenal surgery (RIRS) are stones up to 2cm. In specialized centers, its indication can also be extended to the treatment of larger calculi^2^.

Although RIRS has a lower stone-free rate (SFR) than more invasive procedures such as percutaneous nephrolithotomy, it is less likely to generate lesions, as it does not penetrate the kidney cortex^3^.

One of the advantages of RIRS is the potential to target all parts of the urinary tract, including the renal collecting system. The development of devices with smaller diameters and increased flexibility, associated with a greater deflection angle and an optimized optical system, has increased the possibility of visualizing and treating calculi^5^ ^-^ ^8^. Previous studies have shown that the RIRS SFR ranges from 73.6% to 94.1%^9^.

The classic models of surgical learning become obsolete in the context of the development of new technologies. Minimally invasive surgeries are performed with greater frequency, which creates additional challenges related to their initial, more complex learning curves^10^.

The results of ureteroscopy depend on the availability of equipment and the surgeon’s experience^1^. To get good results, proper training is mandatory. However, the learning curve for RIRS has not yet been well established^11^. Potential outcomes for use in defining the learning curve may include SFR, complication rates, surgical time, fluoroscopy time, radiation doses dispensed, equipment damage, and costs^1^.

Experience in ureteroscopy during residency programs is important for maintaining and developing specific skills. Surgeons with experience in endourology, urologists linked to academic services, and/or the ones graduated for a few years are more likely to use RIRS for the treatment of urinary stones over other surgical techniques. This finding is clearly correlated with their training^1^.

The purpose of this study is to estimate the minimum number of procedures necessary for a surgeon to perform RIRS consistently.

## METHODS

The present study was approved by the Ethics in Research Committee of the University Hospital of the University of São Paulo and was carried out without third-party sponsorship.

A third-year urology resident physician (last year of the residency program in Brazil), who had already performed more than 250 semi-rigid endoscopic ureterolithotripsy procedures, had his first RIRS accompanied by two experienced endourologists. At the time of surgery, one of the endourologists acted as first assistant. The second remained as an observer, outside the surgical field.

An initial cystoscopy was performed in all patients, who were positioned in lithotomy. Two 0.035mm hydrophilic guidewires (ZIPwireTM - Boston Scientific - Marlborough, MA) were inserted through the ureteral meatus and advanced to the renal pelvis with the aid of intraoperative fluoroscopy.

After positioning the guidewires, an 11 or 13FR ureteral sheath (NavigatorTM - Boston Scientific - Marlborough, MA) was inserted up to the level of the renal pelvis. Sheaths of 45cm and 35cm length were used for males and females, respectively. In the Service’s routine, ureteral access sheaths are routinely used in all individuals undergoing RIRS. Patients in whom adequate positioning of the sheath was not possible underwent passage of a double J catheter, with postponement of the surgery. In these cases, the stone was removed after two weeks.

The flexible ureteroscope (Flex-X2S - Karl Storz - Tuttlingen - Germany) was then introduced through the sheath up to the level of the renal pelvis; 0.9% saline solution was used for irrigation. After viewing the calculus, a 200-micron laser fiber was inserted through the device’s working channel. The energy source for fragmentation was a Holmiun 10 w laser producing system (Dornier Medilas^®^ H20 - Germany).

A nitinol stone capture probe (Zero TipTM - Boston Scientific) was used to remove the fragments. Placement of a double J catheter took place after stone treatment in all cases.

All procedures were performed at a single hospital center over one year. If the resident was unable to complete the surgery, the overseeing surgeon would.

A total of 80 surgeries were analyzed by the two experienced endourologists who attended the procedures. The surgeries were divided into 4 groups, according to the order of performance: from the first to the twentieth (Group I), from the twenty-first to the fortieth (Group II), from the forty-first to the sixtieth (Group III), and from the sixty-first to the eightieth (Group IV).

A qualitative analysis was performed using a previously published assessment tool^12^ that encompasses five parameters: tissue handling, bimanual dexterity, depth perception, autonomy, and efficiency (Table 1). Quantitative analysis was performed based on the time required for sheath placement, stone treatment, double-J catheter placement, total ureteral sheath time, and total operative time.

**Table 1:** Global rating scale of the intraoperative assessment tool.

Depth perception^a,b^
1 - Constantly overshoots target, wide swings, slow to correct
2
3 - Some overshooting or missing target, but quick to correct
4
5 - Accurately directs instruments in the correct plane to target
Bimanual dexterity^a,b^
1 - Uses only one hand, ignores non dominant hand, poor coordination between hands
2
3 - Users both hands, but does not optimize interaction between hands
4
5 - Expertly uses both hands in a complementary manner to provide optimal exposure
Efficiency^a,b^
1 - Uncertain, inefficient efforts; many tentative movements; constantly changing focus or persisting without progress
2
3 - Slow, but planned movements are reasonably organized
4
5 - Confident, efficient and safe conduct, maintains focus on task until it is better performed by way of an alternative approach
Tissue handling^a,b^
1 - Rough movements, tears tissue, injures adjacent structures, poor grasper control, grasper frequently slips
2
3 - Handles tissue reasonably well, minor trauma to adjacent tissue (i.e., occasional unnecessary bleeding or slipping of the grasper)
4
5 - Handles tissues well, applies appropriate traction, negligible injury to adjacent structures
Autonomy^a,b^
1. Unable to complete the entire procedure, even in a straightforward case and with extensive verbal guidance
2.
3. Able to complete operation safely with moderate prompting
4.
5. Able to complete operation independently without prompting.

^a^2: middle ground between grades 1 and 3; ^b^4: middle ground between degrees 3 and 5.

**Table 2:** Qualitative analysis.

	TISSUE HANDLING	BIMANUAL DEXTERITY	AUTONOMY	DEPTH PERCEPTION	EFFICIENCY
GROUP I (mean ± SD)	2.65±0.8	2.75±0.5	2.9±1.0	2.8±0.7	2.95± 0.7
GROUP II (mean ± SD)	3.75±0.7	3.75±0.7	4.3±0.8	3.95±0.9	4.15±0.7
GROUP III (mean ± SD)	4.7±0.5	4.75±0.5	5±0	48±0.4	4.85±0.3
GROUP IV (mean ± SD)	4.8±0.4	4.65±0.4	4.95±0.2	4.9±0.3	4.8±0.4
p value	<.0001	<.0001	<.0001	<.0001	<.0001
GROUP I vs GROUP II (p)	<.01	<.01	<.01	<.01	<.01
GROUP I vs GROUP III (p)	<.01	<.01	<.01	<.01	<.01
GROUP II vs GROUP III (p)	<.01	<.01	<.01	<.01	<.01
GROUP I vs GROUP IV (p)	<.01	<.01	<.01	<.01	<.01
GROUP II vs GROUP IV (p)	<.01	<.01		<.01	<.01
GROUP III vs GROUP IV (p)	non-significant	non-significant	non-significant	non-significant	non-significant

**Table 3:** Quantitative analysis.

	SHEATH POSITIONING (MIN)	STONE TREATMENT (MIN)	POSITIONING OF DOUBLE J CATHETER (MIN)	TOTAL URETERAL SHEATH TIME (MIN)	TOTAL SURGICAL TIME (MIN)
GROUP I (mean ± SD)	8.6 ± 3.7	33.95 ±21.4	3.1±1.0	34.2 ±21.4	50.75 ±22.6
GROUP II (mean ± SD)	6.1 ±2.9	24.35 ±17.2	2.45±0.5	24.9 ±16.9	35.3 ±19.4
GROUP III (mean ± SD)	4.35 ±1.1	16.85 ±11.0	3±1.0	18.85 ±10.3	35.3 ±16.6
GROUP IV (mean ± SD)	3.65 ±0.6	24.15 ±10.6	2.4±0.5	27.8±0.6	31.4±9.6
p value	<.0001	0.011	0.012	0.023	0.004
GROUP I vs GROUP II (p)	<.05	<.01	non-significant	non-significant	<.05
GROUP I vs GROUP III (p)	<.01	<.01	non-significant	<.05	<.05
GROUP II vs GROUP III (p)	non-significant	<.01	non-significant	non-significant	non-significant
GROUP I vs GROUP IV (p)	<.01	non-significant	<.05	non-significant	<.01
GROUP II vs GROUP IV (p)	<.05	non-significant	non-significant	non-significant	non-significant
GROUP III vs GROUP IV (p)	non-significant	non-significant	non-significant	non-significant	non-significant

**Table 4:** Postoperative stone-free state.

GROUP I - number of patients (%)	18 (90%)
GROUP II - number of patients (%)	18 (90%)
GROUP III - number of patients (%)	20 (100%)
GROUP IV - number of patients (%)	20 (100%)
p value	>0.999
GROUP I vs GROUP II (p)	non-significant
GROUP I vs GROUP III (p)	non-significant
GROUP II vs GROUP III (p)	non-significant
GROUP I vs GROUP IV (p)	non-significant
GROUP II vs GROUP IV (p)	non-significant
GROUP III vs GROUP IV (p)	non-significant

The groups were evaluated by the Kolmogorov-Smirnov test to confirm a normal distribution. All variables showed normal distribution, being later compared by ANOVA. Afterwards, the Tukey post-test was used for intergroup comparisons.

Two weeks after removal of the double-J catheter, all patients underwent tomography of the total abdomen to evaluate residual lithiasis. Stone-free status was defined as the absence of stones larger than 2mm.

## RESULTS

The mean age of patients was 41 ± 12.5 years, 39 (48.7%) were male and 41 (51.3%) female. There was no difference in stone sizes between groups: 11.4 ± 7.3mm vs. 8.0 ± 3.8mm vs. 11.1 ± 5.2mm vs. 13.7 ± 7.7mm (p=0.12, groups I, II, III, and IV, respectively).

All qualitative variables had significant variation between groups (p<0.001), except between III and IV.

In the quantitative analysis, there was a difference between groups I and IV in the time of placement of the double J catheter (p=0.012). There was an increasing difference in time for sheath placement (p<0.001) and total operative time (p=0.004). The time for stone treatment (p=0.011) was significant only among groups I, II, and III. There was a difference in the total sheath time only between groups I and III (p=0.023).

In the first two groups, only 18 of the 20 patients achieved stone-free status in each of them. In groups 3 and 4, all patients became stone free. There was no significant difference between these rates in any intergroup comparison.

There were no intraoperative complications. In the early postoperative period, two cases of intolerance to the double J catheter were reported in each group and the removal of the double J catheter had to be anticipated, resolving the condition. There were no Clavien III-IV complications. The assistant surgeon did not need to complete the surgery in any case. No equipment damage was observed during the surgeries.

## DISCUSSION

The learning curve is an important issue in surgery^13^. It is considered a representation of a surgeon’s performance improvement over time^1^. The surgical learning curve represents the period when a training surgeon finds the procedure more difficult and takes the longest to complete. There is usually a higher rate of complications and less effectiveness due to inexperience. The point at which the slope of the curve changes or there is no other improvement in performance defines the stage at which technical competence has been reached^14^.

Several attempts have been made to quantify the learning curve for urological procedures, including minimally invasive and endoscopic procedures^10^.

Urological operative technologies are constantly evolving. The number of procedures required to reach the learning curve plateau varied for different procedures and was often affected by experience. In urolithiasis, it is essential to determine the learning curve for each surgical technique. This allows for assessment of surgeons’ progress in training, ensuring competence in each component of the procedure. Before promoting learning in a new technique, it would be imperative to know how many cases a surgeon must perform to be competent in it^1^ ^,^ ^14^.

The surgical treatment of urolithiasis has changed radically in the last 20 years^15^. RIRS refers to the surgical treatment of upper urinary tract pathologies with a retrograde ureteroscopic approach^9^. The concept of endoscopic access to the renal collecting systems for the diagnosis and treatment of diseases of the upper urinary tract was first introduced by V. Marshall, who first described navigation in the renal pelvis with a rudimentary flexible fiberscope, in 1964. Today, RIRS is considered one of the first-line options for active removal of kidney stones^2^.

RIRS consists of a few steps, with many variants proposed in the literature^2^. Recently, the European Urological Association’s guidelines for urolithiasis have shown a broad spectrum of indicators for the active treatment of nephrolithiasis: growing stones, stones in patients at high risk for stone formation, obstructing stones, infections, stones causing pain or hematuria, stones larger than 15mm, patient preference, comorbidity, and social status of individuals in relation to profession or travel^9^.

The effectiveness of RIRS in urolithiasis depends on the surgeon’s experience and on the characteristics of the stone: composition, hardness, number, size, and anatomical location. In recent years, the growth of experience and the refinement of technology have led more surgeons to indicate RIRS to treat larger kidney stones^2^.

The tool we use to assess surgical skills was developed by a Canadian group in 2004. The Global Operative Assessment of Laparoscopic Skills (GOALS) consists of a five-item global rating scale: depth perception (how comfortable the operator works with a monocular optical system, which provides a two-dimensional image on a monitor), bimanual dexterity (optimization of the use of both hands), efficiency (fluidity and progress of the procedure), tissue handling (proper handling of tissues, which includes the adequate use of instruments), and autonomy (surgeon’s technical independence). The tool is viable and reliable^12^.

In our series we were able to verify that a good stone-free rate can be achieved even quickly, but there is still plenty of room for acquisition and refinement of surgical skill and efficiency. In the present study, there were no major complications, even at the beginning of the learning curve, which is also extremely important information.

Cho et al. showed that 56 cases were necessary to reach a plateau in the learning curve. A retrospective review was performed for 100 patients who underwent single-session RIRS. Cases with multiple stones and multiple locations in the same kidney were significant predictors of lower SFR. The cumulative sum analysis curve tended to be flat until the 25^th^ case and showed an increasing pattern but decreased again until the 56^th^ case. After that point, the effectiveness of fragmentation reached a plateau^16^.

Berardinelli et al. showed that the surgeon’s experience influences the RIRS results. A total of 381 surgeries were separated into two groups and a retrospective analysis was performed. In the first group, patients were treated by two surgeons in the initial phase of the learning curve; in the second, the cases were operated on by experienced endourologists. Operative time and general complications were lower in the second group. A non-significant difference was found for SFR15.

Komori et al. reviewed the medical records of 219 patients who underwent RIRS from 2005 to 2013. To compare complications after the introduction of surgery, patients were divided into four groups based on the surgeon’s experience. Complication rates decreased in the more experienced groups. It turned out that around 100 surgeries are needed to reduce serious complications. All complications were reduced, except for urosepsis^17^.

The limitations of our work include not eliminating interpersonal differences. As this study was performed with the evaluation of a single surgeon, it may be difficult to generalize the findings. The assessment tool selected was initially designed for laparoscopy, therefore, it does not consider specific endourology issues, such as irrigation control and use of fluoroscopy. In addition, the sample size calculation is complex, as there are no similar prospective articles to be used as a basis for the calculation.

The stone-free rate remained similar in the four groups, so that the physician’s experience in training did not greatly influence the postoperative results, but with adequate training, surgical results and performance were improved both qualitatively and quantitatively. 

## CONCLUSION

RIRS with flexible instruments for treating kidney stones is a relatively new technique. As there are few studies on its learning curve, more studies are needed to better characterize it.

In our series, after 60 operated cases, all variables did not show additional improvement. Therefore, it appears that 60 cases are a reasonable estimate of the experience needed for the RIRS learning curve to plateau.
